# Accidente cerebrovascular isquémico en un paciente con HIV: neurosífilis y síndrome antifosfolipídico

**DOI:** 10.7705/biomedica.7830

**Published:** 2026-06-01

**Authors:** Sebastián Ayala, María del Mar Ortiz, María Carolina Vásquez

**Affiliations:** 1. Departamento de Medicina Interna, Facultad de Salud, Universidad del Valle, Cali, Colombia Departamento de Medicina Interna Facultad de Salud Universidad del Valle Cali Colombia; 2. Grupo Interinstitucional de Medicina Interna 1 (GIMI 1), Departamento de Medicina Interna, Universidad Libre, Cali, Colombia Grupo Interinstitucional de Medicina Interna 1 (GIMI 1) Departamento de Medicina Interna Universidad Libre Cali Colombia; 3. Departamento de Hospitalización, Clínica Imbanaco, Cali, Colombia Departamento de Hospitalización Clínica Imbanaco Cali Colombia

**Keywords:** VIH, accidente cerebrovascular, sífilis, neurosífilis, síndrome antifosfolípido, anticuerpos antifosfolípidos, HIV, stroke, syphilis, neurosyphilis, antiphospholipid syndrome, antibodies, antiphospholipid

## Abstract

La infección por HIV es un factor de riesgo para el desarrollo de enfermedades cardiovasculares, incluyendo los accidentes cerebrovasculares isquémicos.

Se presenta el caso de un hombre de 27 años con sida, con un accidente cerebrovascular isquémico de la arteria cerebral media izquierda. Los exámenes de laboratorio confirmaron el diagnóstico de neurosífilis y, además, confirmaron la existencia de anticuerpos antifosfolípidos. Siete meses después del tratamiento de la neurosífilis, de la anticoagulación y de la terapia antirretroviral, se confirmó la persistencia de anticuerpos antifosfolípidos. El enfoque diagnóstico de un accidente cerebral vascular en personas con HIV es un reto, ya que puede asociarse a múltiples etiologías. La relevancia de los anticuerpos antifosfolípidos en estos pacientes es tema de debate y debe tenerse en cuenta según el cuadro clínico.

## Abstract (en)

HIV infection is a risk factor for the development of cardiovascular diseases including ischemic strokes, for which there are different multiple etiologies in these patients. We present the case of a 27-year-old man with AIDS, with an ischemic stroke in the left middle cerebral artery territory. Laboratory studies confirmed the diagnosis of neurosyphilis but also the existence of antiphospholipid antibodies. Seven months after the treatment of neurosyphilis, anticoagulation and antiretroviral therapy, the persistence of antiphospholipid antibodies was confirmed.

The diagnostic approach to a vascular stroke in people with HIV is a challenge since there may be multiple etiologies. The relevance of antiphospholipid antibodies in these patients is a matter of debate and should be considered depending on the clinical scenario.

La infección por el virus de la inmunodeficiencia humana (HIV, *human immunodeficiency virus*) aumenta el riesgo de presentar eventos isquémicos cerebrales. En diversos estudios se ha señalado que este riesgo puede ser hasta dos veces mayor en las personas infectadas por el HIV en comparación con aquellas no infectadas ^1^^,^^2^. La incidencia de los accidentes cerebrovasculares aumenta con la edad (5 por 1000 al año entre los 18 y los 29 años y 17 por 1000 al año entre los 60 y los 70 años) (5 y 17 por 1000 al año, entre los 18 y los 29 y entre los 60 y los 70 años de edad, respectivamente), lo cual indica que otros factores de riesgo adquiridos durante la vida potencian el riesgo cardiovascular, además de la infección del virus ^3^^,^^4^.

A medida que disminuye el número de linfocitos T CD4+ o aumenta la carga viral, se incrementa el riesgo de desarrollar eventos isquémicos, ya que tener un conteo de linfocitos T CD4+ menor de 200 por pl, se reconoce como el factor más importante ^5^.

Entre las etiologías más comunes en los pacientes con HIV, se encuentran la vasculopatía asociada al HIV (20 al 32 %), las infecciones oportunistas o neoplasias (13 al 28 %), la embolia cardiaca (5 al 15 %) y las coagulopatías (19 al 49 %) ^6^. En comparación, en los pacientes negativos para HIV, aproximadamente el 75 % de los casos de eventos isquémicos cerebrales se deben a embolia cardiaca, aterosclerosis de grandes arterias o enfermedad de pequeños vasos ^7^^,^^8^.

El abordaje diagnóstico adecuado permite hacer un diagnóstico diferencial certero y encontrar etiologías infrecuentes en estos casos.

Se presenta el caso de un hombre con infección por HIV en estadio A3 sin tratamiento, que presenta un accidente cerebrovascular isquémico en la arteria cerebral media izquierda y en quien se documentan dos posibles causas: sífilis meningovascular y síndrome de anticuerpos antifosfolípidos.

## Presentación del caso

Se trata de un hombre de 27 años, mestizo, originario y procedente del suroccidente colombiano, con antecedentes de infección por HIV diagnosticado cuatro años antes del ingreso, sin recibir terapia antirretroviral. El paciente consultó al servicio de urgencias por un cuadro clínico consistente en la pérdida de la consciencia de 20 minutos de duración, que lo llevó a tener una caída desde su propia altura que le provocó una laceración en el hombro derecho. No presentó movimientos anormales, pérdida del control de esfínteres ni desviación de la mirada.

Entre los antecedentes, refirió un adenoma parotídeo derecho (confirmado por biopsia en el 2018) sin seguimiento y síntomas de depresión (insomnio, anhedonia, afecto triste y pérdida subjetiva de la memoria de dos años de evolución). Negó consumir medicamentos o sustancias psicoactivas.

**Figura 1:**
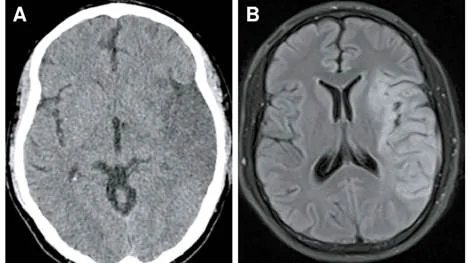
A) Tomografía simple de cráneo, ventana de cerebro, imagen axial a nivel de los tálamos. Se evidencia lesión hipodensa en el territorio de la arteria cerebral media del lado izquierdo, con pérdida de la diferenciación corticosubcortical y borramiento de surcos cerebrales, por edema citotóxico. No hay desplazamiento de la línea media. B) Resonancia simple de cerebro, secuencia FLAIR, imagen axial a nivel de los núcleos de la base. Se evidencia área hiperintensa en el territorio de la arteria cerebral media del lado izquierdo, comprometiendo la ínsula, el giro precentral, el giro poscentral y el lóbulo parietal inferior. La lesión se extiende a la sustancia blanca profunda y el brazo anterior de la cápsula interna. Hay leve efecto de masa con desplazamiento de la línea media.

Sus signos vitales eran normales; sin embargo, presentó un examen neurológico alterado por afasia motora y sensorial, sin compromiso de los nervios craneales ni alteraciones motoras ni sensitivas, sin signos cerebelosos y con una marcha normal. Se le practicó una tomografía craneal simple en la que se observó hipodensidad en el territorio de la arteria cerebral media izquierda, concordante con un accidente cerebrovascular isquémico. En la resonancia magnética, se constataron signos indicativos de un evento isquémico subagudo en el territorio de la arteria cerebral media izquierda (figura 1).

Según el tiempo de evolución y las características imagenológicas sugestivas de un infarto, el paciente no era candidato a trombólisis ni manejo endovascular. En los estudios iniciales de laboratorio, se encontró linfopenia (linfocitos totales de 910/mm^3^) en el hemograma, función renal y hepática normales, serologías para hepatitis viral (A, B, C) negativas, linfocitos T CD4+ de 142 células/mm^3^ (15 % del total), carga viral de HIV de 10 234 copias/ml (log 4,01), prueba treponémica (FTA-ABS) reactiva y prueba no treponémica por reagina plasmática rápida reactiva en dilución de 1:256.

En la angiorresonancia se identificó un mínimo flujo intracraneal en la arteria cerebral media izquierda -concordante con un infarto cerebral reciente- e hipoplasia del segmento V4 del sistema vértebro-basilar como variante anatómica. No hubo hallazgos evidentes ni indirectos de vasculitis.

Es importante señalar que la ausencia de signos de vasculopatía en la angiorresonancia no descarta el compromiso vascular, dado que esta modalidad tiene una sensibilidad limitada para detectar vasculitis. El rendimiento diagnóstico mejora con la angiotomografía cerebral y, especialmente, con la angiografía digital, considerada el método más sensible, aunque invasivo.

La angiorresonancia tiene una sensibilidad entre el 62 y el 79 % y una especificidad del 83 al 87 % para detectar vasculitis intracraneal; estos valores pueden aumentar hasta el 96 o el 100 % al emplear secuencias 3D-ToF en equipos de tres tesla.

Por otra parte, la angiotomografía alcanza una sensibilidad del 97 % y una especificidad del 99 % para estenosis del 50 % o menos. Finalmente, la angiografía digital continúa siendo el método más sensible (hasta el 94 %) y específico (hasta el 97 %), pese a requerir un procedimiento invasivo ^9^^-^^11^.

En otros estudios, como el electrocardiograma de doce derivaciones, la radiografía de tórax, el ecocardiograma transesofágico y el Holter electrocardiográfico, no se observaron alteraciones. Teniendo en cuenta los hallazgos anatómicos y de laboratorio, y dado que el paciente presentaba una serología en sangre con prueba no treponémica reactiva en dilución 1:256 y FTA-ABS igualmente reactiva, se practicaron punciones lumbares seriadas con las cuales se confirmó el diagnóstico de sífilis (cuadro 1).

**Cuadro 1:** Resultados de punciones lumbares seriadas

Característica	Punción lumbar 1	Punción lumbar 2	Punción lumbar 3
Presión de apertura	12 cm H_2_0	11 cm H_2_0	12 cm H_2_0
Aspecto del LCR	Ligeramente turbio	Transparente	Transparente
Hematíes/mm^3^	65	4	2
Polimorfonucleares/mm^3^	36	0	0
Linfocitos/mm^3^	37	61	74
Mononucleares/mm^3^	0	0	0
Glucosa (mg/dl)	58	30	42
Proteínas totales (mg/dl)	68	74	73
LDH (U/L)	73	130	86
VDRLen LCR	Reactivo	Reactivo	Reactivo
Gram para bacterias	Negativo	Negativo	Negativo
Cultivo para bacterias	Negativo	Negativo	Negativo
Baciloscopia en LCR	Negativo para BAAR	Negativo para BAAR	Negativo para BAAR
Cultivo de *Mycobacterium* spp.	Negativo	Negativo	Negativo
PCR para *M. tuberculosis*	No detectado	No detectado	No detectado
Tinta china	Negativo	Negativo	Negativo
Antígeno contra *Cryptococcus* spp.	Negativo	-	-

LCR: líquido cefalorraquídeo; BAAR: bacilos ácido-alcohol resistentes; LDH: lactato deshidrogenasa; PCR: *polymerase chain reaction* ; VDRL: *Venereal Disease Research Laboratory*

La sintomatología neurológica, junto con las imágenes sugestivas de infarto isquémico, motivaron el estudio del líquido cefalorraquídeo, el cual mostró pleocitosis, hipoglucorraquia, proteinorraquia y una prueba no treponémica reactiva, hallazgos clásicos que confirmaron el diagnóstico clínico-microbiológico de sífilis meningovascular. Se inició tratamiento con penicilina cristalina en dosis de 4 millones de unidades internacionales cada 4 horas por 14 días.

Ante la ausencia de signos de vasculopatía en la angiorresonancia, se decidió ampliar la búsqueda de coagulopatías (cuadro 2), encontrándose anticuerpos anticardiolipina IgM y anti-beta 2 glicoproteína 1 IgM e IgG, por lo que se planteó el diagnóstico de síndrome de anticuerpos antifosfolípidos y se inició anticoagulación plena con enoxaparina para, posteriormente, iniciar tratamiento con warfarina.

**Cuadro 2:** Laboratorios al ingreso y control a los siete meses de iniciado el tratamiento

Laboratorio	Ingreso	Control
ANA	Positivo de 1/160, patrón granular fino	Positivo de 1/160, patrón granular fino
C3 (mg/dl)	131	121
C4 (mg/dl)	24	26
Anticuerpos anti DNAds	Negativo	Negativo
Anti-RO (Ul/ml)	7,6 (negativo)	1,4 (negativo)
Anti-LA (Ul/ml)	2,4 (negativo)	1,5 (negativo)
Anti-RNP (Ul/ml)	1,3 (negativo)	3,6 U (negativo)
Anti-SM (Ul/ml)	2,4 (negativo)	2,8 U (negativo)
Anti-β2GP I IgM (U/ml)	< 9,37 U (positivo > 20)	< 9,37 U (positivo > 20 U)
Anti-β2GP I IgG (U/ml)	0,0 (positivo > 20)	0,0 U
Anti-cardiolipina IgM (MPl)	93,9 (positivo > 20)	77,5 MPL (positivo > 20)
Anti-cardiolipina IgG (GPl)	1,6 (negativo)	0,0 GPL (negativo)
Anticoagulante lúpico	No realizado	RE: 1,26 (positivo > 1,2)
Veneno de víbora de Russell	No realizado	Razón de 1,02 (negativo)

ANA: anticuerpos antinucleares; Anti-β2GP I: anticuerpos contra la beta-2 glucoproteína I; anti- DNAds: anticuerpos contra el ADN de doble cadena; anti-LA: anticuerpos contra el antígeno La/ SSB; anti-RNP: anticuerpos contra la ribonucleoproteína; anti-RO: anticuerpos contra el antígeno Ro/SSA; anti-SM: anticuerpos contra el antígeno Smith; C3: complemento 3; C4: complemento 4; GPI: unidades de fosfolípidos IgG; IgG: inmunoglobulina G; IgM: inmunoglobulina M; MPI: unidades de fosfolípidos IgM (*IgM phospholipid units*); RE: razón de escrutinio

Si bien la presencia de anticuerpos antifosfolípidos fue persistente a lo largo del seguimiento y el diagnóstico cumplía con los criterios clínico-inmunológicos, es importante resaltar que la decisión de iniciar anticoagulación en un paciente con un evento isquémico único y una etiología clara como la sífilis meningovascular, no está completamente respaldada por la literatura.

El síndrome de anticuerpos antifosfolípidos pudiera ser un hallazgo incidental en este contexto; sin embargo, ante la persistencia serológica y el cuadro clínico, se optó por instaurar anticoagulación indefinida como medida preventiva.

Además, al paciente se le dio de alta con terapia antirretroviral consistente en emtricitabina-tenofovir-atazanavir-ritonavir. En el control ambulatorio a los siete meses del egreso, el paciente presentaba mejoría de la afasia, linfocitos T CD4+ de 320 células/mm^3^, carga viral indetectable y persistencia de anticuerpos antifosfolípidos positivos (cuadro 2), por lo que se continuó la anticoagulación de manera indefinida.

### Consideraciones éticas

Este reporte de caso tuvo en cuenta las normas vigentes sobre investigación en seres humanos, contempladas en la Declaración de Helsinki de la Asociación Médica Mundial y la Resolución 8430 de 1993 del Ministerio de Salud de Colombia. De igual manera, se obtuvo el consentimiento informado del paciente para la elaboración de la historia clínica, la recolección de datos e imágenes fotográficas y la posterior publicación científica.

## Discusión

Las personas infectadas con HIV cuentan con un mayor riesgo vascular, lo que favorece la aparición de eventos isquémicos cerebrales ^12^. Ante las diversas causas que pueden explicar los fenómenos isquémicos en estos pacientes, se han planteado algoritmos que permitan hacer un adecuado diagnóstico diferencial de enfermedades como infecciones oportunistas, embolia cardiaca, vasculitis, arterioesclerosis, coagulopatía, autoinmunidad y enfermedad de pequeños vasos ^6^.

En el caso presentado, la primera posibilidad diagnóstica fue la neurosífilis de tipo meningovascular, teniendo en cuenta el compromiso isquémico en el territorio de la arteria cerebral media izquierda y la serología positiva en el líquido cefalorraquídeo.

Se ha descrito que, aunque la incidencia de sífilis ha disminuido en la era posterior a la penicilina, la neurosífilis ha resurgido asociada con la epidemia de la infección por HIV ^13^. La bacteria *Treponema pallidum* tiene la habilidad de generar aneurismas micóticos, fenómenos vasculíticos y estados de hipercoagulabilidad, que pueden llevar a eventos isquémicos ^14^.

Con frecuencia, se ha descrito que la sífilis meningovascular produce un grado de arteriopatía oclusiva concéntrica y segmentaria que se correlaciona con los hallazgos de tipo «rosario» en los estudios angiográficos a nivel de la arteria cerebral media ^14^^,^^15^. Por otro lado, algunos autores han confirmado que, aunque los estudios angiográficos pueden revelar diversos grados de arteriopatía, no son absolutos y no existen hallazgos específicos para la sífilis meningovascular, por lo que la clínica, sumada a los antecedentes del paciente y a los estudios del líquido cefalorraquídeo, es lo que debe orientar hacia el diagnóstico ^16^^,^^17^. Por esta razón, al no tener hallazgos conclusivos en la angiografía, en este caso se puede determinar que es probable que la neurosífilis sea la causa del fenómeno isquémico.

Se han planteado múltiples discusiones frente a lo significativa que puede ser la serología antifosfolipídica positiva en un paciente con infección por HIV, ya que para algunos puede ser parte de un fenómeno inmunológico sin repercusiones trombóticas ^18^^-^^21^, mientras que para otros puede ser la causa de algunos eventos isquémicos arteriales en esta población ^22^^-^^24^.

En varios estudios, se ha planteado que los pacientes con HIV tienen más riesgo de desarrollar anticuerpos anticardiolipina (IgM e IgG) en comparación con los pacientes sin presencia del virus e, incluso, que el riesgo puede ser aún mayor que en los pacientes con lupus eritematoso sistémico ^25^.

El determinar si la presencia de anticuerpos antifosfolípidos en los pacientes con HIV tiene o no un efecto trombogénico, aún es tema de estudio ^26^^,^^27^. Cabe resaltar que en este caso, comparado con otros reportes, se logró documentar la persistencia de los anticuerpos antifosfolípidos siete meses después del evento, a pesar de que, para esa fecha, se había logrado el control virológico (CD4 = 320 células/mm^3^ y una carga viral indetectable), lo cual es importante debido a que se ha reportado que los títulos de los anticuerpos antifosfolípidos disminuyen o, incluso, desaparecen con el control de la viremia ^19^. Dicho hallazgo sugiere que, en este paciente, sí existe un síndrome de anticuerpos antifosfolípidos coexistente y con relevancia clínica.

## Conclusiones

Existen múltiples causas de los eventos cerebrovasculares en las personas que padecen HIV, con frecuencias diferentes en comparación con las de los pacientes sin el virus. Aunque el virus por sí mismo aumenta la incidencia de los eventos isquémicos, se requiere un enfoque diagnóstico organizado para determinar de manera adecuada su etiología (únicos o múltiples). La neurosífilis debe considerarse como un posible agente etiológico a pesar de la ausencia de los hallazgos tradicionales en la neuroimagen.

La determinación etiológica del evento isquémico en este paciente representa un reto clínico complejo. La sífilis meningovascular es una causa probable, teniendo en cuenta el cuadro clínico, la serología positiva en el líquido cefalorraquídeo y los hallazgos indicativos de infarto isquémico.

Aunque la angiorresonancia no mostró signos de vasculitis, esto no excluye el diagnóstico, ya que técnicas más sensibles, como la angiografía digital cerebral o la imagen de alta resolución de la pared vascular, han demostrado mayor utilidad en la detección de la vasculitis infecciosa, incluso, cuando la angiorresonancia convencional es normal ^28^.

Por otro lado, la presencia persistente de anticuerpos antifosfolípidos (en particular, la IgM anticardiolipina y la anti-β2 glicoproteína I), plantea la posibilidad de un síndrome de anticuerpos antifosfolípidos clínicamente relevante. Aunque se han reportado casos en los que estos anticuerpos aparecen transitoriamente en casos de infecciones crónicas como el HIV, también se ha documentado su papel en eventos trombóticos, especialmente cuando persisten a pesar del control virológico ^29^^,^^30^.

En este sentido, la coexistencia de ambas condiciones (neurosífilis y síndrome de anticuerpos antifosfolípidos) podría haber contribuido sinérgicamente al desarrollo del accidente cerebrovascular isquémico.

Con este caso se destaca la importancia de una evaluación diagnóstica amplia y personalizada de los pacientes con infección por HIV, considerando no solo causas infecciosas, sino también, fenómenos autoinmunitarios como el síndrome de anticuerpos antifosfolípidos.
